# Preoperative assessment using a scoring system after neoadjuvant chemotherapy with docetaxel, cisplatin, and 5-fluorouracil for locally advanced esophageal cancer: Who can avoid surgery?

**DOI:** 10.1371/journal.pone.0328835

**Published:** 2025-08-08

**Authors:** Yasunori Kurahashi, Toshihiko Tomita, Takuya Okugawa, Kazuhiro Kitajima, Motoki Murakami, Shugo Kohno, Yudai Hojo, Eiichiro Nakao, Tatsuro Nakamura, Yoshinori Ishida, Shinichiro Shinzaki, Hisashi Shinohara

**Affiliations:** 1 Department of Gastroenterological Surgery, Hyogo Medical University, Hyogo, Japan; 2 Department of Gastroenterology, Faculty of Medicine, Hyogo Medical University, Hyogo, Japan; 3 Department of Radiology, Hyogo Medical University, Hyogo, Japan; All India Institute of Medical Sciences, INDIA

## Abstract

Neoadjuvant chemotherapy (NAC) with docetaxel, cisplatin, and 5-fluorouracil (DCF) is highly effective for advanced esophageal squamous cell carcinoma, and patients with a good response sometimes desire to avoid surgery. The aim of this single-center, retrospective study was to predict the probability of residual tumors using a newly devised scoring system and identify conditions that may obviate the need for surgery. Between January 2017 and March 2024, 106 patients received NAC with DCF, followed by radical resection at our institution. After NAC, patients underwent multimodal assessments to correlate the evaluation parameters with residual tumors. A scoring system was developed by incorporating the parameters that exhibited significant differences. After calculating the scores for all patients, a receiver operating characteristic (ROC) curve was generated to determine the optimal cutoff value for predicting residual tumors. Eighteen patients achieved a pathological complete response, of whom 12 showed complete tumor disappearance, including lymph node metastases. The scoring system included the following seven parameters: endoscopic irregularity, elevation, and pink-color sign after iodine staining; identification of the main tumor, regional lymph nodes ≥5 mm in long diameter, presence of positive lymph node findings on computed tomography; and positive 18F-fluorodeoxyglucose uptake on positron emission tomography. The area under the ROC curve was 0.957, with a cutoff value of 3 for residual tumors. All patients with a score of 0 showed complete tumor disappearance. Our scoring system suggests that surgery might be safely omitted in patients with a score of 0. Comprehensive and flexible clinical decision-making is essential.

## Introduction

Esophageal cancer, one of the most aggressive gastrointestinal malignancies, is the seventh leading cause of cancer and the sixth leading cause of cancer-related deaths worldwide [1]. Multidisciplinary treatment combining surgery, chemotherapy, and radiation therapy is the mainstay of treatment for advanced esophageal cancer. According to the JCOG 1109 NExT study in Japan, neoadjuvant chemotherapy (NAC) with three courses of docetaxel, cisplatin, and 5-fluorouracil (DCF), followed by radical resection, has become the standard treatment for locally advanced resectable esophageal cancer [2]. This regimen is highly effective, with a pathological complete response (pCR) rate of 19.8% and a 3-year overall survival rate of 72.1%. Furthermore, for locally advanced unresectable esophageal cancer, the JCOG 1510 TRIANgLE trial is currently verifying the effectiveness of induction DCF therapy followed by conversion surgery [3]. The use of this regimen in the preoperative setting for esophageal cancer is expected to increase.

When preoperative therapy is effective, patients sometimes wish to avoid surgery. Many studies have compared active surveillance with surgery for patients who have achieved complete clinical response (cCR) after neoadjuvant chemoradiotherapy [4–9]. Although active surveillance is not a standard alternative to surgery and is used selectively for patients with esophageal cancer who refuse surgery or are medically unfit for major surgery after completion of neoadjuvant chemoradiotherapy [4,5], some studies have reported comparable outcomes between active surveillance and surgery in patients with cCR [7,8]. It is reasonable to consider avoiding surgery in patients who have achieved cCR after NAC with DCF, which is highly effective as monotherapy and has a relatively high pCR rate. Developing a reliable method for predicting pCR would be advantageous for patients.

In the current 12th edition of the Japanese Classification of Esophageal Cancer, the effectiveness of preoperative treatment is evaluated according to the Response Evaluation Criteria In Solid Tumors (RECIST) classification [10–12]. However, this evaluation fails to represent the status of esophageal cancer after preoperative treatment accurately. For example, lymph nodes with a short axis of 10 mm or less are deemed negative, yet metastatic lymph nodes of a similar size are often observed. Additionally, pCR has been observed among the cases judged as non-CR/non-PD (remarkable response [RR]) on endoscopic evaluation. As a result, it is very difficult to decide whether to undergo or avoid surgery based on the criteria recommended in the Japanese Classification of Esophageal Cancer.

In the present study, we aimed to evaluate cancer lesions after NAC with DCF by examining esophagogastroduodenoscopy (EGD), computed tomography (CT), and positron emission tomography (PET/CT) to predict the probability of residual tumors using a newly devised scoring system based on our own criteria and identify conditions that may obviate the need for surgery.

## Materials and methods

### Patients

This single-center, retrospective study was conducted to predict the probability of residual tumors after NAC with DCF. Between January 2017 and March 2024, 200 consecutive patients with esophageal squamous cell carcinoma underwent esophagectomy at the Department of Surgery, Hyogo Medical University, Hyogo, Japan. Of these, 106 patients who received NAC with DCF followed by radical resection were included in this study. This study was approved by the local ethics committee of the Hyogo Medical University (approval number 4832), and comprehensive informed consent or substitute for it was obtained from all patients. Access to the clinical data began on November 1, 2024. The data were anonymized after collection, and since then, access to information that could identify individual participants has become impossible.

### NAC with DCF

NAC with DCF was administered according to the JCOG1109 NExT trial regimen [2]. Docetaxel (70 mg/m²) and cisplatin (70 mg/m²) were administered on day 1, and 5-fluorouracil (750 mg/m²/day) was administered on days 1–5. This cycle was repeated every 21 days for a total of three cycles. The dosage was appropriately adjusted based on the severity of the adverse events and the patient’s condition. If necessary, chemotherapy was discontinued midway, and surgical treatment was performed.

### Surgical treatment

In principle, surgery was performed within 56 days from day 5 of the last course of NAC with DCF. The patients underwent subtotal esophagectomy with either 2-field or 3-field lymph node dissection via right thoracotomy or video-assisted thoracic surgery. A laparoscopic approach was used for abdominal lymph node dissection and gastric mobilization, except in patients with a history of abdominal surgery. Reconstruction using the jejunum or colon was performed via open surgery.

### Pathological examination

In accordance with the 12th edition of the Japanese Classification of Esophageal Cancer, a diagnosis of pCR was made when no viable cancer cells were observed in the primary tumor during postoperative pathological examination. Among the cases diagnosed as pCR, those without lymph node metastasis and with complete disappearance of cancer cells (ypT0N0M0) were defined as “pCR/N-,” whereas those with pCR as primary tumor and observed lymph node metastasis were defined as “pCR/N+.”

### Preoperative assessment and scoring system

After NAC, we assessed cancer lesions using multiple imaging modalities, including preoperative EGD, cervicothoracic contrast-enhanced CT (1 mm thickness, SOMATOM Definition Edge Plus, Siemens AG, Munich, Germany), and PET/CT (Discovery IQ, GE Healthcare, Waukesha, WI, USA). Subsequently, we verified the relationship between each evaluation parameter and the presence of residual cancer cells using statistical analysis. The evaluation parameters were as follows: biopsy results from EGD; endoscopic findings of stenosis, white moss, ulceration or mucosal depression, irregularity, and elevation; presence of an unstained area and pink-color sign after iodine staining; identification of the main tumor on CT; regional lymph nodes measuring ≥5 mm and ≥10 mm in long diameter, except for the flattened subcarinal lymph nodes that were clearly not metastases; the presence of contrast effects indicative of lymph node metastasis (such as ring enhancement, strong enhancement, and feathering); positive PET/CT findings (presence of fluorodeoxyglucose [FDG] uptake that was higher than the mediastinal blood pool activity on visual inspection by radiologists) [13–16]; and elevated tumor markers (squamous cell carcinoma antigen >2.5 ng/mL). The judgment was reached by consensus between two surgeons who were blinded to the pathological results after thoroughly reviewing all accessible endoscopic and radiological reports. The relationship between the positivity of these parameters and residual tumors was evaluated, and parameters showing significant differences were incorporated into the scoring system. Scores were calculated for each of the 106 cases. A receiver operating characteristic (ROC) curve was subsequently generated to assess the ability of the scoring system to predict residual tumors. Based on this analysis, a cutoff value was derived to indicate the presence of residual tumors. Additionally, a score indicating no residual tumor was determined, establishing a threshold below which surgery could be safely omitted.

### Statistical analysis

Recurrence-free survival (RFS) was analyzed using the Kaplan–Meier method and compared using the log-rank test. A comparison was made between the presence of findings for each parameter and the presence of residual cancer cells, and sensitivity and specificity were calculated. P-values for each parameter were calculated using Fisher’s exact test for categorical variables, with a significance level of 5% (two-sided). An ROC curve was constructed to compare the calculated scores with the presence of residual tumors, and the area under the curve (AUC) was calculated. The accuracy of the test increased with AUC values. The AUC values were interpreted as follows: AUC = 0.50 indicated random prediction; 0.60–0.70, poor validity; 0.70–0.80, fair validity; 0.80–0.90, good validity; and >0.90, excellent validity. The optimal cutoff value was determined at the inflection point of the ROC curve. All statistical analyses were performed using JMP Pro software (version 17.0; SAS Institute Inc., Cary, NC, USA). Statistical significance was set at P < 0.05.

## Results

### Characteristics of the 106 patients and the result of NAC

The clinical characteristics of the patients are summarized in Table 1. The median age (range) was 70 (41–84) years, and there were 79 males and 27 females. All patients were histologically diagnosed with esophageal squamous cell carcinoma using EGD before treatment initiation. The results of NAC with DCF are shown in Table 2. Most patients received three courses of DCF; however, 26.4% received only one or two courses due to adverse events. One-third of the patients experienced grade 4 neutropenia, and one-quarter experienced grade 4 febrile neutropenia.

**Table 1:** Patient characteristics.

Variable	n = 106
Age median (range)	70 (41-84)
Sex	
Male/ Female	79/ 27
cT factor*	
1a/ 1b/ 2/ 3r/ 3br/ 4	1/ 6/ 18/ 40/ 39/2
cN factor*	
0/ 1/ 2/ 3	14/ 48/ 38/ 6
cM factor*	
0/ 1a/ 1b	90/ 15/ 1
cStage*	
II/ IIIA/ IIIB/ IVA/ IVB	14/ 50/ 39/ 2/ 1
Tumor localization*	
Ce/ Ut/ Mt/ Lt/ Jz	4/ 18/ 41/ 38/ 5

* The 12th edition of the Japanese Classification of Esophageal Cancer.

Ce, cervical esophagus; Ut, upper thoracic esophagus; Mt, middle thoracic esophagus; Lt, lower thoracic esophagus; Jz, zone of the esophagogastric junction.

**Table 2:** Results of Neoadjuvant Chemotherapy with DCF.

Number of courses	n = 106	%
1	11	10.4
2	17	16.0
3	77	72.6
4	1	0.9
Grade 4 neutropenia		
Yes	35	33.0
No	71	67.0
Febrile neutropenia		
Yes	26	24.5
No	80	75.5
Therapeutic effect (tumor)		
grade 0	1	0.9
grade 1a	33	31.1
grade 1b	17	16.0
grade 2	37	34.9
grade 3	18	17.0
LN metastasis of grade 3 cases	n = 18	
positive (pCR/N+)	6	33.3
negative (pCR/N-)	12	66.7

LN, lymph node; pCR, pathological complete response; pCR/N + , pCR with residual lymph node metastasis; pCR/N-, pCR without lymph node metastasis.

### Postoperative pathological examination and RFS of pCR cases

**Fig 1:**
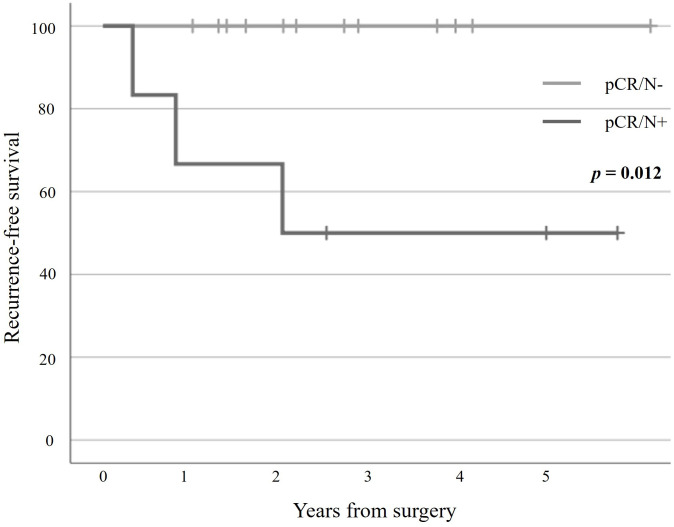
Recurrence-free Survival Curves for 18 Patients who achieved Complete Response (pCR). “pCR/N+” and “pCR/N-” represent “pCR with residual lymph node metastasis” and “pCR without lymph node metastasis,” respectively. All 12 pCR/N- patients survived without recurrence, with a median survival time of 29.6 months.

Postoperative pathological examination revealed that 18 patients (17.0%) showed complete disappearance of cancer cells in the primary tumor, which was diagnosed as pCR. Among these, six patients (33.3%) had lymph node metastasis (pCR/N+), whereas the remaining 12 patients (66.7%) showed complete disappearance of all cancer cells, including those in the lymph nodes (pCR/N-). All 12 pCR/N- patients survived without recurrence, with a median survival time of 29.6 months. In contrast, among the six pCR/N+ patients, three had recurrence, and one died of the primary disease 19 months after surgery. The RFS curves are shown in Fig 1.

### Relationship between each evaluation parameter and the presence of residual tumor

The relationship between each evaluation parameter and the presence of residual tumors is presented in Table 3. Because a positive biopsy clearly indicated the presence of cancer cells, the sensitivity and specificity for residual tumors of each parameter other than the biopsy were calculated, and a univariate analysis was performed. Significant differences were observed between the presence of residual tumors and the following parameters: irregularity, elevation, presence of an unstained area and pink-color sign after iodine staining in endoscopic findings; identification of the main tumor on CT; regional lymph nodes measuring ≥5 mm and ≥10 mm in long diameter; presence of contrast effects indicative of lymph node metastasis; and presence of FDG uptake on PET/CT.

**Table 3:** Relationship between each Evaluation Parameter and the Presence of Residual Tumor.

Evaluation parameter	Sensitivity	Specificity	p value
EGD			
Stenosis	17.1	77.8	0.7358
White moss	18.2	88.9	0.7318
Ulceration or mucosal depression	46.6	72.2	0.1936
Irregularity	88.6	72.2	<0.0001
Elevation	88.6	55.6	0.0001
Unstained area and pink-color sign after iodine staining	81.0	88.9	<0.0001
CT			
Identification of the main tumor	48.9	100.0	<0.0001
Regional lymph nodes (long diameter) ≥10 mm	42.2	82.9	0.0099
Regional lymph nodes (long diameter) ≥5 mm	90.6	31.7	0.0151
Ring enhancement, strong enhancement, and feathering	46.9	100.0	<0.0001
PET/CT			
FDG uptake	71.8	100.0	0.0111
Tumor marker			
Serum SCC antigen≥2.5 ng/mL	3.2	100.0	1.0000

EGD, esophagogastroduodenoscopy; CT, computed tomography; PET/CT, positron emission tomography; FDG, fluorodeoxyglucose; SCC, squamous cell carcinoma.

Significant evaluation parameters used in the scoring system have been underlined.

### Scoring system

**Fig 2:**
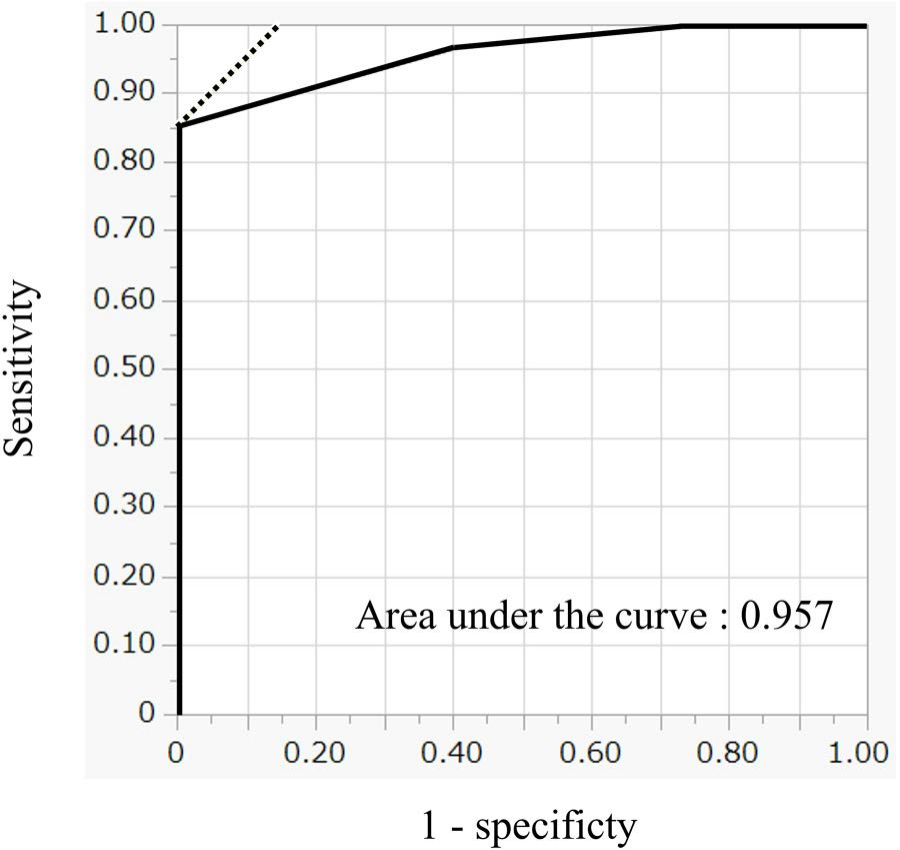
ROC Curve for Residual tumor Prediction by the Scoring System. The ROC curve closely follows the left-hand border and top border of the ROC space. The area under the curve was 0.957, indicating excellent validity of the test.

Using the above seven parameters (underlined in Table 3), we scored positive findings as 1 point each, with a maximum score of 7 points. For the long diameter of lymph nodes, we adopted a stricter criterion of ≥5 mm. The results of the scoring system are presented in Table 4. All patients with a score of 0 showed complete tumor disappearance. The rate of tumor disappearance decreased as the score increased, and no tumor disappearance was observed in patients with a score of 3 or higher. The ROC curve for residual tumor prediction using the scoring system is shown in Fig 2. The AUC under the ROC curve was 0.957, indicating test validity. According to the ROC analysis, the optimal cutoff for residual tumors was 3. At this cutoff, the sensitivity and specificity were 100% and 85.1%, respectively.

**Table 4:** Results of the Scoring System.

Score	pCR/N-	pCR/N+	non-pCR	% of total tumor disappearance
0	3	0	0	100.0
1	4	0	3	57.1
2	5	3	11	26.3
3	0	3	19	0.0
4	0	0	22	0.0
5	0	0	17	0.0
6	0	0	14	0.0
7	0	0	8	0.0

pCR, pathological complete response; pCR/N + , pCR with residual lymph node metastasis; pCR/N-, pCR without lymph node metastasis.

## Discussion

In Japan, according to the JCOG 1109 NExT study, NAC with three courses of DCF followed by radical resection has become the standard treatment for resectable, locally advanced esophageal squamous cell carcinoma [2]. As the DCF regimen is highly effective, we sometimes encounter patients who wish to avoid surgery after a significant response. Some studies have reported comparable outcomes between active surveillance and surgery for patients with cCR after neoadjuvant chemoradiotherapy for esophageal cancer [7,8]. In contrast, other studies have reported superior overall survival, disease-specific survival, and RFS for cCR patients who underwent surgery to those under surveillance [17,18]. Surgeons are justified in recommending surgery for these patients. However, if the postoperative pathology confirms pCR, it could be argued that surgery might have been unnecessary, particularly in older or high-risk patients. Therefore, it is advantageous to explore methods that can accurately predict pCR and thereby safely circumvent surgery. In this study, we sought to predict the likelihood of residual tumor presence using a new scoring system derived from endoscopic, CT, and PET/CT findings post-NAC with DCF, aiming to assess the feasibility of avoiding surgery in responsive cases.

One problem with the preoperative evaluation used in the current 12th edition of the Japanese Classification of Esophageal Cancer is the definition of a negative lymph node as having a short diameter of 10 mm or less on CT, because metastatic lymph nodes of this size are frequently encountered [10,11]. In this study, lymph nodes with a long diameter of ≥5 mm and ≥10 mm were considered positive parameters, and we adopted the stricter criterion of ≥5 mm for our scoring system. Although the number of false positives is likely to increase, it seems reasonable to set a more stringent criterion to safely avoid surgery. Additionally, lymph nodes with findings that strongly suggest metastasis, such as ring enhancement, strong enhancement, and feathering, were highly likely to be metastasis-positive; therefore, these findings were also incorporated into the evaluation criteria. Another issue is the endoscopic evaluation criteria in the Japanese Classification. They identify only entirely flat lesions as cCR, with the exception of minor granulation, and do not consider the pink-color sign during iodine staining. In our study, endoscopic observations of mucosal depression and ulceration did not show a significant correlation with the presence of cancer remnants. Moreover, six out of the 26 cases classified as non-CR/non-PD (RR) by these criteria were found to be pCR in our series (23.0%; data not shown). The pink-color sign, characterized by a notable color shift from whitish yellow to pink within 2–3 minutes after iodine application, has been documented to correlate highly with the diagnosis of high-grade esophageal intraepithelial neoplasia and invasive cancer [19,20]. In the present study, we defined and evaluated novel parameters based on the observation items in the Japanese Classification of Esophageal Cancer to address the limitations of existing classifications and better align with our specific research objectives.

Postoperative pathological examination and RFS of pCR cases suggest that only pCR/N- cases were considered potential candidates for avoiding surgery. In our scoring system, the cutoff value for residual tumor was set at 3, implying a cutoff value of 2 for tumor disappearance. However, overlooking residual tumors is unacceptable when considering the option of safely avoiding surgery. As shown in Table 4, the only score associated with no residual tumor was 0, suggesting that surgery might be avoidable if the biopsy result is negative and the score is 0. Although pCR/N- could be accurately predicted in only 25% of cases (3 of 12 patients with a score of 0), we believe that strict criteria are essential to prevent missing residual tumors. Based on these findings, the following clinical decision-making process is proposed: For patients with a score of 3 or more, surgery is strongly recommended. For patients with a score of 2 or less, the possibility of tumor disappearance should be discussed. For those with a score of 0, the option of avoiding surgery could be proposed.

When surgery is circumvented based on the scoring system, radiation therapy or chemoradiotherapy serves as an alternative option to ensure safety. Although radiation therapy may suffice for patients presenting only a local tumor remnant, chemoradiotherapy is generally favored if the patient’s condition permits, owing to the unreliability of lymph node diagnosis. Although chemoradiotherapy could potentially relax the criteria for avoiding surgery, establishing the appropriate irradiation field continues to pose a significant challenge.

In this study, we developed an original scoring system to identify the conditions under which surgery can be safely avoided. Nevertheless, accurate predictions proved challenging, and we cannot confidently recommend avoiding surgery even for patients with a score of 0. Completing treatment with planned surgery remains the primary objective. From the perspective of informed consent, however, the final decision regarding the treatment ultimately rests with the patient. Presenting the score calculated using our scoring system can facilitate more informed decision-making when evaluating whether to undergo or avoid surgery.

The limitations of this study include its retrospective, single-center design and small sample size. Additionally, some data on iodine staining and PET/CT were missing. However, these omissions often resulted from the lack of detailed examinations in cases where residual tumor was evident in other multiple parameters. Although this could lead to underestimation in high-scoring regions, it is believed that this did not significantly impact the prediction of pCR. In the future, we plan to conduct a prospective study to validate this scoring system by comprehensively assessing all scoring parameters and increasing the sample size.

In conclusion, we investigated the feasibility of avoiding surgery in patients who exhibited a significant response to NAC with DCF. Our newly developed scoring system suggests that surgery may be safely omitted if the biopsy result is negative and the score is 0. Although prospective validation is indispensable for confirmation, comprehensive and flexible clinical decision-making that considers individual patient circumstances and preferences remains crucial.

## Supporting information

S1 Dataset. (XLSX)

## References

[1] Sung H, Ferlay J, Siegel RL, Laversanne M, Soerjomataram I, Jemal A, et al. Global Cancer Statistics 2020: GLOBOCAN Estimates of Incidence and Mortality Worldwide for 36 Cancers in 185 Countries. CA Cancer J Clin. 2021;71(3):209–49. doi: 10.3322/caac.21660 33538338

[2] Kato K, Machida R, Ito Y, Daiko H, Ozawa S, Ogata T, et al. Doublet chemotherapy, triplet chemotherapy, or doublet chemotherapy combined with radiotherapy as neoadjuvant treatment for locally advanced oesophageal cancer (JCOG1109 NExT): a randomised, controlled, open-label, phase 3 trial. The Lancet. 2024;404(10447):55–66. doi: 10.1016/s0140-6736(24)00745-1 38876133

[3] Terada M, Hara H, Daiko H, Mizusawa J, Kadota T, Hori K, et al. Phase III study of tri-modality combination therapy with induction docetaxel plus cisplatin and 5-fluorouracil versus definitive chemoradiotherapy for locally advanced unresectable squamous-cell carcinoma of the thoracic esophagus (JCOG1510: TRIANgLE). Jpn J Clin Oncol. 2019;49(11):1055–60. doi: 10.1093/jjco/hyz112 31411696

[4] Noordman BJ, Wijnhoven BPL, Lagarde SM, Biermann K, van der Gaast A, Spaander MCW, et al. Active surveillance in clinically complete responders after neoadjuvant chemoradiotherapy for esophageal or junctional cancer. Dis Esophagus. 2017;30(12):1–8. doi: 10.1093/dote/dox100 28881890

[5] van der Wilk BJ, Eyck BM, Spaander MCW, Valkema R, Lagarde SM, Wijnhoven BPL, et al. Towards an Organ-Sparing Approach for Locally Advanced Esophageal Cancer. Dig Surg. 2019;36(6):462–9. doi: 10.1159/000493435 30227434 PMC6878756

[6] Valkema MJ, van der Wilk BJ, Eyck BM, Wijnhoven BPL, Spaander MCW, Doukas M, et al. Surveillance of Clinically Complete Responders Using Serial 18F-FDG PET/CT Scans in Patients with Esophageal Cancer After Neoadjuvant Chemoradiotherapy. J Nucl Med. 2021;62(4):486–92. doi: 10.2967/jnumed.120.247981 32887759 PMC8049375

[7] van der Wilk BJ, Noordman BJ, Neijenhuis LKA, Nieboer D, Nieuwenhuijzen GAP, Sosef MN, et al. Active Surveillance Versus Immediate Surgery in Clinically Complete Responders After Neoadjuvant Chemoradiotherapy for Esophageal Cancer: A Multicenter Propensity Matched Study. Ann Surg. 2021;274(6):1009–16. doi: 10.1097/SLA.0000000000003636 31592898

[8] van der Wilk BJ, Eyck BM, Hofstetter WL, Ajani JA, Piessen G, Castoro C, et al. Chemoradiotherapy Followed by Active Surveillance Versus Standard Esophagectomy for Esophageal Cancer: A Systematic Review and Individual Patient Data Meta-analysis. Ann Surg. 2022;275(3):467–76. doi: 10.1097/SLA.0000000000004930 34191461

[9] De Pasqual CA, Weindelmayer J, Gervasi MC, Torroni L, Pavarana M, Cenzi D, et al. Active surveillance for clinical complete responders after chemoradiotherapy for oesophageal squamous cell carcinoma. Br J Surg. 2024;111(2):znae036. doi: 10.1093/bjs/znae036 38415879

[10] Mine S, Tanaka K, Kawachi H, Shirakawa Y, Kitagawa Y, Toh Y, et al. Japanese Classification of Esophageal Cancer, 12th Edition: Part I. Esophagus. 2024;21(3):179–215. doi: 10.1007/s10388-024-01054-y 38568243 PMC11199297

[11] Doki Y, Tanaka K, Kawachi H, Shirakawa Y, Kitagawa Y, Toh Y, et al. Japanese Classification of Esophageal Cancer, 12th Edition: Part II. Esophagus. 2024;21(3):216–69. doi: 10.1007/s10388-024-01048-w 38512393 PMC11199314

[12] Eisenhauer EA, Therasse P, Bogaerts J, Schwartz LH, Sargent D, Ford R, et al. New response evaluation criteria in solid tumours: revised RECIST guideline (version 1.1). Eur J Cancer. 2009;45(2):228–47. doi: 10.1016/j.ejca.2008.10.026 19097774

[13] Karashima R, Watanabe M, Imamura Y, Ida S, Baba Y, Iwagami S, et al. Advantages of FDG-PET/CT over CT alone in the preoperative assessment of lymph node metastasis in patients with esophageal cancer. Surg Today. 2015;45(4):471–7. doi: 10.1007/s00595-014-0965-6 24969050

[14] Kaida H, Yasuda T, Shiraishi O, Kato H, Kimura Y, Hanaoka K, et al. The usefulness of the total metabolic tumor volume for predicting the postoperative recurrence of thoracic esophageal squamous cell carcinoma. BMC Cancer. 2022;22(1):1176. doi: 10.1186/s12885-022-10281-4 36376801 PMC9664655

[15] Wahl RL, Siegel BA, Coleman RE, Gatsonis CG, PET Study Group. Prospective multicenter study of axillary nodal staging by positron emission tomography in breast cancer: a report of the staging breast cancer with PET Study Group. J Clin Oncol. 2004;22(2):277–85. doi: 10.1200/JCO.2004.04.148 14722036

[16] Kitajima K, Fukushima K, Miyoshi Y, Katsuura T, Igarashi Y, Kawanaka Y, et al. Diagnostic and prognostic value of (18)F-FDG PET/CT for axillary lymph node staging in patients with breast cancer. Jpn J Radiol. 2016;34(3):220–8. doi: 10.1007/s11604-015-0515-1 26715510

[17] Ohkura Y, Shindoh J, Ueno M, Iizuka T, Udagawa H. Comparison of Outcome of Esophagectomy Versus Nonsurgical Treatment for Resectable Esophageal Cancer with Clinical Complete Response to Neoadjuvant Therapy. Ann Surg Oncol. 2018;25(8):2428–33. doi: 10.1245/s10434-018-6437-2 29564587

[18] Piessen G, Messager M, Mirabel X, Briez N, Robb WB, Adenis A, et al. Is there a role for surgery for patients with a complete clinical response after chemoradiation for esophageal cancer? An intention-to-treat case-control study. Ann Surg. 2013;258(5):793–9; discussion 799-800. doi: 10.1097/SLA.0000000000000228 24096755

[19] Ishihara R, Yamada T, Iishi H, Kato M, Yamamoto S, Yamamoto S, et al. Quantitative analysis of the color change after iodine staining for diagnosing esophageal high-grade intraepithelial neoplasia and invasive cancer. Gastrointest Endosc. 2009;69(2):213–8. doi: 10.1016/j.gie.2008.04.052 18718584

[20] Zheng J-Y, Chen Y-H, Chen Y-Y, Zheng X-L, Zhong S-S, Deng W-Y, et al. Presence of pink-color sign within 1 min after iodine staining has high diagnostic accordance rate for esophageal high-grade intraepithelial neoplasia/invasive cancer. Saudi J Gastroenterol. 2019;25(2):113–8. doi: 10.4103/sjg.SJG_274_18 30588952 PMC6457187

